# Chromatin Immunoprecipitation (ChIP): Revisiting the Efficacy of Sample Preparation, Sonication, Quantification of Sheared DNA, and Analysis via PCR

**DOI:** 10.1371/journal.pone.0026015

**Published:** 2011-10-25

**Authors:** Pamela D. Schoppee Bortz, Brian R. Wamhoff

**Affiliations:** 1 Cardiovascular Division, Department of Medicine, University of Virginia Health System, Charlottesville, Virginia, United States of America; 2 Robert M. Berne Cardiovascular Research Center, University of Virginia Health System, Charlottesville, Virginia, United States of America; National Cancer Institute, United States of America

## Abstract

The “quantitative” ChIP, a tool commonly used to study protein-DNA interactions in cells and tissue, is a difficult assay often plagued with technical error. We present, herein, the process required to merge multiple protocols into a quick, reliable and easy method and an approach to accurately quantify ChIP DNA prior to performing PCR. We demonstrate that high intensity sonication for at least 30 min is required for full cellular disruption and maximum DNA recovery because ChIP lysis buffers fail to lyse formaldehyde-fixed cells. In addition, extracting ChIP DNA with chelex-100 yields samples that are too dilute for evaluation of shearing efficiency or quantification via nanospectrophotometry. However, DNA extracted from the Mock-ChIP supernatant via the phenol-chloroform-isoamyl alcohol (PCIA) method can be used to evaluate DNA shearing efficiency and used as the standard in a fluorescence-based microplate assay. This enabled accurate quantification of DNA in chelex-extracted ChIP samples and normalization to total DNA concentration prior to performing real-time PCR (rtPCR). Thus, a quick ChIP assay that can be completed in nine bench hours over two days has been validated along with a rapid, accurate and repeatable way to quantify ChIP DNA. The resulting rtPCR data more accurately depicts treatment effects on protein-DNA interactions of interest.

## Introduction

Molecular biologists commonly use the chromatin immunoprecipitation (**ChIP**) assay is a tool to study protein-DNA interactions in healthy and diseased biological systems. As a result, numerous variations of the original approach to ChIP (reviewed by [1]) are present within the peer-reviewed literature (recent examples: [2]-[11]) and on molecular biology protocol websites. Generally, the assay method contains: 1) a fixation step designed to fuse the protein-DNA interactions of interest in place; 2) a series of cell lysis steps intended to separate and wash away extraneous cellular components (cellular membranes, cytoplasmic proteins and RNA) while retaining the nuclear chromatin compartment; 3) a form of chromatin fragmentation, be it mechanical or enzymatic; 4) a mode of antibody-protein-chromatin complex precipitation (protein A/G coated-agarose or magnetic beads); and finally, 5) a precipitated DNA purification step [12]. Once the ChIP sample is generated, real-time polymerase-chain reaction (**rtPCR**) is typically used to quantify the relationship of interest; hence, the “quantitative” ChIP assay. Most methods include protease inhibitors in the immunoprecipitation (**IP**) buffer and, depending on the targets of interest, some also include phosphatase or other specific enzymatic inhibitors [4], [7]. The assay can be cumbersome and fraught with ample opportunity to introduce technical error. When the assay fails, it can be very difficult to determine why. As a result, many attempts to perform the ChIP assay are met with extreme frustration or complete failure and the quality of the ChIP data in the literature varies substantially.

The objectives of this work were two-fold: the first was to merge several published ChIP protocols, including many from our lab and from our colleagues [7], [9], [13]-[16] into a quick, reliable and easy method. Our hypothesis was that controlling the quality of the chromatin preparation would yield the greatest chance for a successful outcome (assuming appropriate antibodies were available for the immunoprecipitation phase of the assay) so we focused our attention on the efficacy of the cell harvest, cell lysing and washing, and DNA fragmentation steps. The result was a validated Quick ChIP protocol that could be completed in nine bench hours over two days. Our second objective was to identify an accurate way to quantify sheared DNA in the final ChIP sample so that when rtPCR was performed the samples were normalized to DNA concentration. Because rtPCR results are typically normalized to total DNA controls, our hypothesis was that this would eliminate experimental artifacts and/or unmask experimental differences that might otherwise be lost should rtPCR be performed on samples or total DNA controls of varying concentrations. We accomplished this by demonstrating that PicoGreen dsDNA dye can be used to accurately quantify sheared DNA in ChIP samples when a similarly prepared sheared DNA sample is used as the reference standard.

## Materials and Methods

### Cell culture

Rat aortic smooth muscle cells (**SMC**, passages 15 – 18) were the primary source of chromatin used during the development and validation of the protocols presented herein. They were cultured on 150 mm dishes in DMEM:F12 media (Gibco) supplemented with 10% fetal bovine serum (Hyclone), 1% antibiotics (100X penicillin/streptomycin solution, Gibco) and L-glutamine (1.6 mM, Gibco) until they approached 70 – 95% confluency [14], [17]. For experiments designed to determine whether the method could be used to repeat previously published results [16], [18], SMCs were growth arrested at 60% confluency for 72 h in insulin- and serum-free media further supplemented with ascorbic acid (3.52 µg/ml), apo-transferrin (5.0 µg/ml) and sodium selenite (6.25 ng/ml; supplements purchased from Sigma) then treated for 24 h with platelet-derived growth factor-BB (30 ng/ml; **PDGF-BB**, Millipore). Chromatin from primary cultures of human vascular endothelial cells (**VEC**) was used for some experiments. These cells were isolated from umbilical cords and cultured by our collaborators [19].

### Ethics statement

Our collaborator's [19] procurement of discarded human umbilical cord tissue, with written, informed consent from anonymous donors for the purpose of performing scientific research at the University of Virginia, was approved by Martha Jefferson Hospital's Institutional Review Board.

### Cell fixation and harvest for the ChIP assay

**Figure 1:**
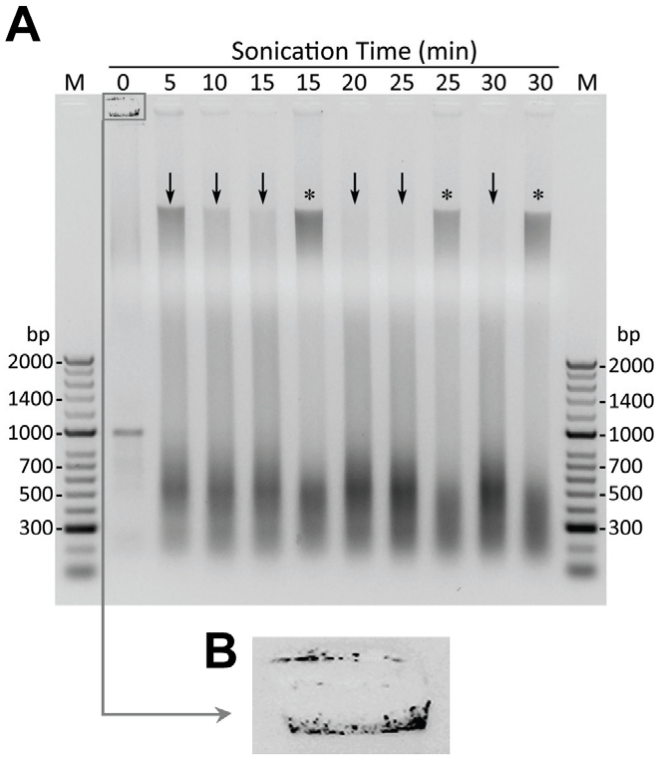
Effect of Sonication Time on Degree of Chromatin Shearing in Fresh or Frozen Cell Suspensions. Fixed rat SMC (10^7^ cells/ml) were sonicated in 5 min intervals from 5 to 30 min then reverse cross-linking was performed before DNA was extracted using PCIA. Final DNA concentrations were determined via nanospectrophotometry; 2 µg of DNA per time point was used to evaluate the effect of shearing by electrophoresis in a 1% agarose gel (**A**). A small volume (7.5 µl) of unsonicated, unextracted sample (0 min) was also electrophoresed for comparison (**M**: a 100 base-pair DNA marker). Sonication was either performed immediately (**↓**) or performed after the samples had been frozen at –80°C for two weeks (*). For fresh samples, increased sonication time decreased the amount of high molecular weight DNA and increased the amount of DNA accumulating in the 400 – 600 base pair region. In contrast, when samples were frozen then sonicated, a high molecular weight fraction of DNA persisted through 30 min indicating that freezing the sample altered the DNA's susceptibility to shearing by sonication. Magnification of the well containing the ‘0 min’ sample component that failed to migrate into the gel (**B**) shows a punctuate pattern on both the positive and negative side of the well suggesting genomic DNA was retained in intact nuclei.

At the time of cell harvest, culture confluency percentage was estimated then compared to historical data in order to estimate the number of cells per dish (see Appendix S1). Formaldehyde (37%) was diluted to 1% in the culture media then cells were incubated at 37°C for 10 min. Fixation was terminated at room temperature with 125 mM Glycine (Sigma) for 5 min. Hereafter, the cells, chromatin preparations and ChIP reactions where handled at 4°C. For initial experiments (Figure 1 and Figure S1) cells were harvested according to an established protocol [13]-[16]. Briefly, cells were scraped from culture dishes in Dulbecco's phosphate buffered saline (**DPBS**; Gibco) containing a complete protease inhibitor cocktail (**cPI**; Roche Diagnostics), washed with DPBS plus cPI then washed in a buffer containing nonidet-P40 (0.5%, **NP40**) plus cPI to lyse cells before being resuspended in a buffer containing sodium dodecyl sulfate (1%, **SDS**) for the sonication step. Formaldehyde and detergents were purchased from Fisher Scientific. To reduce the complexity of the ChIP assay, cells in all subsequent experiments were harvested as described [7]. They were washed once in DPBS then harvested by scrapping in cold DPBS (without protease inhibitors). The cells were pelleted by centrifugation (2000 *g*, 5 min, 4°C) and washed twice with cold IP buffer (50 mM Tris, pH 7.5, 150 mM NaCl, 5 mM EDTA, 0.5% NP40, 0.1% triton-X 100; **TX100**) containing 1.5% of a protease/phosphatase inhibitor solution (**PPI**, Pierce “Halt”) before the final dilution in preparation for sonication.

### DNA shearing by sonication

A Bioruptor™ XL sonicator (Diagenode) was used to shear chromatin because it could shear 24 samples per run; an aspect that can improve the uniformity of sample shearing across treatments. Per the manufacturer's recommendation, a layer of ice was maintained in the water bath at all times during the sonication process. The protocol recommended in the Bioruptor XL instruction manual (15 bursts of 30 sec ON and 30 sec OFF; e. g. 15 min) was used to determine whether cell density would affect the degree of chromatin shearing (see Appendix S1). Additional sample handling and DNA sonication conditions were determined as discussed (see “Sonication Conditions” under Results and Discussion). Immediately following sonication, the samples were cleared by centrifugation (12,000 *g*, 10 min, 4°C); supernatants containing the sheared chromatin were transferred to new tubes, pooled (by treatment when appropriate) and mixed, then aliquoted for either DNA extraction (sonication optimization experiments) or for ChIP reactions (typically 185 – 200 µl each; the approximate DNA equivalent of two million cells) and Total DNA controls (typically 50 µl combined with 150 µl of 100% ethanol) before being frozen at –20°C to –80°C for later use.

### Chromatin immunoprecipitation and extraction with chelex-100

For a given experiment, chromatin preparations from each treatment were designated as specific target (**AB–IP1…** ***n***) or control reactions containing either rabbit anti-mouse RNA polymerase II (**Pol–IP**; 5 µg; Santa Cruz Biotechnologies, #sc-899X), or non-immune rabbit IgG (e. g. species matched **Mock–IP**; 5 µg; Millipore #PP64); the positive and negative controls, respectively. For the purpose of assay validation, the antibodies used in specific target reactions were: rabbit anti-human serum response factor (**SRF**, 5 µg; Santa Cruz Biotechnologies #sc-13029X) and rabbit anti- Tetrahymena acetylated histone 4 (**AcH4**, 5 µg; Millipore #06-598). ChIP reactions were brought up to 400 µl with IP+PPI buffer, incubated on a rotating platform for 18 h at 4°C then cleared by centrifugation (12,000 *g*, 30 sec) before the supernatant (containing the antibody-protein-chromatin complexes) was transferred to tubes containing Protein A agarose beads blocked with salmon sperm DNA (40 µl of a 50% slurry; Millipore). The immunoprecipitation tubes were incubated with rotation for 2 h at 4°C after which the antibody-bead complexes were pelleted by centrifugation and the supernatant was discarded. The beads were washed five times with 1 ml of cold IP buffer without PPI.

ChIP and Total DNA (ethanol-precipitated from 50 µl of each starting chromatin preparation) was purified at room temperature using chelex-100 as described [7]. In brief, 100 µl of a 10% chelex-100 resin solution (w:v, Bio-Rad Laboratories) was added to the tubes containing antibody-protein-chromatin-agarose bead pellets; the tubes were boiled at 95°C for 10 min with mixing (1000 rpm) then cooled to 65°C so that a proteinase K (Roche Diagnostics) digestion could be performed. The samples were centrifuged (12,000 *g*, 30 sec, 4°C) to pellet the agarose beads and resin after heat inactivation of proteinase K at 85°C; supernatants containing the immunoprecipitated chromatin were transferred to new tubes. Ultra pure RNase- DNase-free water (80 µl; Invitrogen) was added to the bead/resin pellet, boiled as above, then cooled and centrifuged before the final supernatant was removed and combined with the first supernatant.

### Extraction of sheared DNA using Phenol:Chlorofom:Isoamyl alcohol (PCIA)

Formaldehyde-fixed chromatin samples were incubated at 65°C for at least 6 h with mixing at 1000 rpm using a Thermomixer R (Eppendorf) to reverse cross-linking [20]. Cooled samples were brought up to 500 µl with IP buffer (for ease of handling) then mixed 1∶1 with PCIA (Fisher Scientific) at room temperature for 10 min before the organic and aqueous phases were separated via centrifugation (14,000 rpm, 10 min, 23°C). The aqueous phase was transferred to a new tube containing an equal volume of chloroform (Fisher Scientific); after mixing, the organic and aqueous phases were separated by centrifugation at 4°C as above. The aqueous phase was transferred to a new tube containing approximately 2.75X volumes of 100% ethanol and sodium acetate diluted to 75 mM (pH 7.0 – 8.0; Fluka) then frozen at –20°C overnight. The DNA was precipitated by centrifugation (14,000 rpm, 5 min, 4°C) then the DNA pellet was washed with 70% ethanol, dried under a vacuum, reconstituted in TE buffer (10 mM Tris, 1 mM EDTA, pH 7.5 – 8.0) and stored at –20°C for later use as the reference standard in the PicoGreen DNA assay.

### Quantification of ChIP DNA

Concentrations of the final ChIP and Total DNA samples were determined in triplicate: 5 µl of sample, 95 µl of TE buffer without dye and 100 µl of TE buffer containing PicoGreen® dsDNA dye (Invitrogen) diluted 1∶200 were combined in a solid white 96-well microplate (Costar) and incubated at room temperature for 5 min before a FLUOStar Omega (BMG Labtech) microplate reader equipped with MARS Data analysis software (BMG Labtech) was used to read the plate. Dye excitation was set at 485 nm and emission fluorescence was measured at 520 nm according to the kit protocol. Occasionally, Total DNA samples were diluted 1∶25 in RNase- DNase-free water, pH≥8.0, before they were assayed. The concentration of the PCIA-extracted sheared DNA stock solution used to create the reference standard dilution series (against which unknown ChIP samples were compared) was determined using a NanoDrop 1000 spectrophotometer (Thermo Fisher Scientific). See Appendix S2 and Figure S2 for validation of PicoGreen® dye use with sheared DNA from fixed SMC; PicoGreen assay data representing two chromatin preparations are provided in Table S1.

### Quantitative real-time PCR

Real-time PCR was performed in triplicate using quantified (1 – 2 ng) or unquantified (6.25 µl) ChIP or Total DNA in a 25 µl reaction containing 1X iQ™ SYBR® Green supermix (Bio-Rad Laboratories) and 400 nM each of forward and reverse primers. The sheared DNA used as a standard in the PicoGreen assay was further diluted and used to generate a standard curve in each real-time PCR assay from which assay efficiencies and sample ‘starting quantities’ (**SQ**) were determined. The data are analyzed using SQ for the specific target, positive control or negative control ChIP samples divided by SQ for the Total DNA control and presented as relative enrichment. We chose to calculate enrichment based on SQ values rather than using the ΔC*_T_* method because the results were virtually identical and the calculation was less complicated. To test our ChIP assay, a region of the smooth muscle α-actin (*ACTA2*) promoter (bases –47 to –193 containing SRF DNA binding elements; e. g. CArG boxes: [CC(A/T)_6_GG] [21]), was amplified from SMC chromatin (see Figure S3) using published primers [14], [16], [18].

### Evaluation of sheared DNA via agarose gel electrophoresis

Extracted DNA samples were loaded (either 20 µl or 2 µg of total DNA/lane) in a 1% agarose gel (Bio-Rad Laboratories) containing 0.01% ethidium bromide (Fisher Scientific). A 100 base-pair DNA marker (Bioline) was used for size comparison. Electrophoresis was performed at room temperature for 90 min at 95 volts. Gel images were captured in color using the UVP DigiDoc-It image acquisition system (Fisher Scientific) then converted to black and white and inverted using Photoshop CS2 (Adobe).

### Evaluation of sonicated cells by microscopy

Cells (2.5 µl) suspended in IP buffer (containing 0.5% NP40 and 1% TX100) were dropped onto glass slides and stored at 4°C under humid conditions. On the following day, samples were examined using phase-contrast light microscopy (Nikon Eclipse TS100 microscope equipped with an Olympus QColor 3 digital camera) at 40X, 100X or 200X magnification. Samples were kept moist during imaging with DPBS. Images were captured in color using QCapture Pro51 imaging software (QImaging™) then converted to black and white using Photoshop CS2 (Adobe).

## Results and Discussion

### Sonication conditions

Identifying and optimizing appropriate conditions for shearing DNA by sonication required an understanding of how cell density, sonication time, sonication intensity, and freezing formaldehyde-fixed cells affected cell integrity, DNA shearing efficiency and total DNA recovery. We did not evaluate the effect of changing the sonication pulse parameters.

Numerous ChIP protocols recommend shearing DNA at a starting density of 10^7^ cells/ml; however, when cultured cells are treated with growth stimulants or inhibitors over time, the number of cells harvested per treatment varies. Thus, to standardize a sonication protocol that would be effective across multiple cell densities, it was important to understand how it affected DNA shearing efficiency. To evaluate the effect of cell density on sonication, the density of a single large preparation of formaldehyde-fixed SMC was estimated (see Appendix S1) then a series of diluted cell suspensions was generated in 300 µl of lysis buffer (the maximum volume recommended). The diluted samples, along with an equal volume of undiluted cell suspension, were sonicated simultaneously then the samples were subjected to cross-linking reversal, DNA extraction using the PCIA protocol and gel electrophoresis as described in the methods. Within the range tested (approximately 1.0 – 3.7×10^6^ cells/300 µl), no affect of cell density was observed (Figure S1). We concluded that a uniform sample volume could be used for all treatments within a given experiment if the treatment that resulted in the greatest number of cells was used to determine the final sample volume at a target density of 107 cells/ml.

The next step toward identifying parameters that would enable us to generate a consistent and high quality chromatin preparation was performing a time course analysis of DNA shearing that included freshly prepared and frozen cell samples. To do this, a suspension of fixed SMC was equally divided and sonicated in 5 min intervals from 5 to 30 min (a small volume of unsonicated sample was retained for later comparison). Sonication was either performed immediately (6/9 tubes) or performed after the samples had been frozen and stored at –80°C (3/9 tubes). As expected, increased sonication time decreased the amount of high molecular weight DNA and increased the amount of DNA accumulating in the targeted region of 400 – 600 base pairs in freshly sonicated samples (Figure 1A, arrows). In contrast, a high molecular weight fraction of DNA persisted through 30 min of sonication in samples that had been frozen and thawed once before sonication (Figure 1A, asterisks). Importantly, this indicates that freezing formaldehyde-fixed SMC alters the DNA's susceptibility to shearing. Hereafter, cell suspensions were sonicated before being frozen.

**Figure 2:**
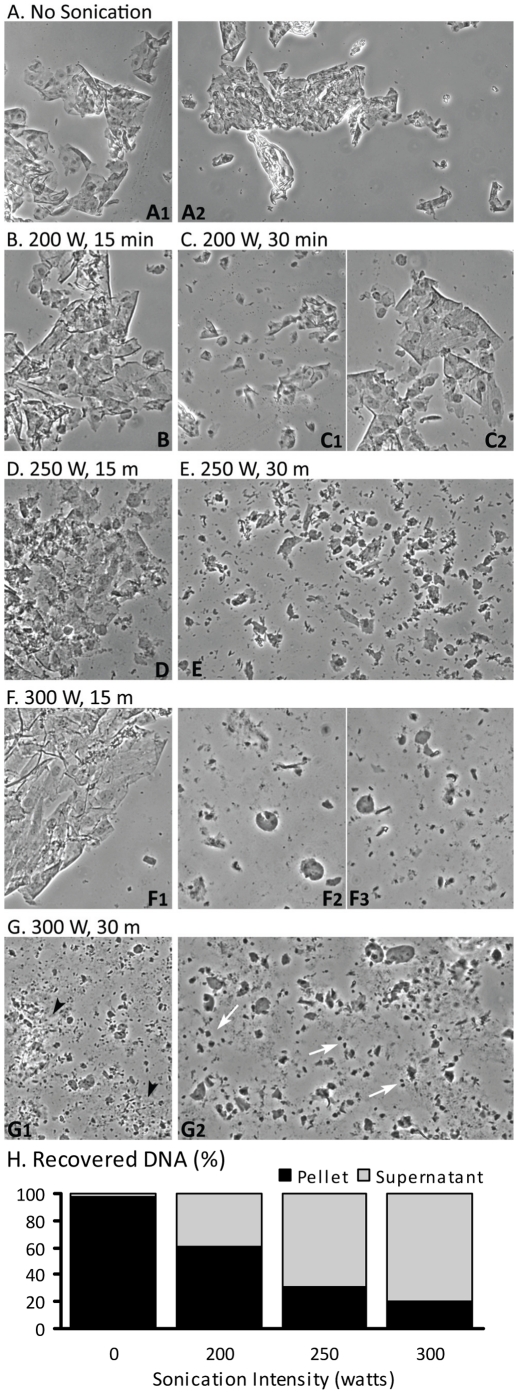
Effect of Sonication Intensity and Time on Cell Integrity and DNA Recovery from Rat SMC. Fixed cells suspended in IP+PPI buffer were spotted onto slides (2.5 µl) before sonication (**A**), or after being sonicated for 15 or 30 min at 200, 250 and 300 watts (W; **B** – **G2**). Samples were viewed with phase-contrast light microscopy; images were captured using QCapture Pro51 imaging software (Magnifications: **A1**, **C1**,: 40X; **A2**, **B**, **C2**, **D** – **F1**, **G1**: 100X; **F2**, **F3**, **G2**: 200X). After sample clearing by centrifugation, DNA in the supernatant and debris pellet was extracted via PCIA from an unsonicated sample (0 min) or from samples sonicated for 30 min at each intensity; DNA concentrations were determined using a NanoDrop then graphed as a percentage of the total recovered per sonication intensity level (**H**). Unsonicated SMC remained intact in large sheets even after sitting in IP buffer for more than 24 h. Sonication at 200 W for 15 min had no effect on the cell sheets (**B**) but after 30 min (**C1**, **C2**) individual cells and some nuclei were present. At 250 W, sonication for 15 min damaged some cell sheets resulting in visible debris (**D**); after 30 min, more cell damage was apparent but many nuclei and some individual cells remained intact (**E**). After 15 min of sonication at 300 W, some cell sheets were still present (**F1**) but the free nuclei displayed more damage (**F2**, **F3**). Sonication for 30 min at 300 W (**G1**, **G2**) pulverize the cell sheets and most nuclei; clumps of cellular debris (**G1**, black arrowheads) and intact nucleoli (**G2**, white arrows) as well as a few intact nuclei were visible. Centrifugation of unsonicated cells (‘0’ intensity) resulted in nearly a complete loss of DNA in the pellet fraction (**H**) but as the intensity of sonication increased the percentage of DNA present in the supernatant fraction also increased.

Upon further examination of the gel lane containing a portion of the original unsonicated, unprocessed cell suspension (Figure 1A, 0 min) it became apparent that the sample component that migrated into the gel had a banding pattern more reminiscent of rRNA than of high molecular weight DNA. We had observed this previously when sonicated samples were electrophoresed without being subjected to cross-linking reversal or DNA extraction (data not shown). Because processing the samples eliminated the rRNA-like bands, experiments were not preformed to further address this issue. Another portion of the sample, presumably the high molecular weight genomic DNA, failed to migrate into the gel at all (Figure 1B). This component had a punctate appearance present on both the positive and negative side of the well, which suggested it was chromatin retained within intact nuclei. To confirm this, we dropped 2.5 µl of fixed, unsonicated SMC suspended in IP buffer (which contains NP40 and TX100 but not SDS) [7] onto a glass slide and examined it via phase light microscopy. We expected to see intact nuclei and cellular debris but were very surprised to discover the predominant component of the suspension was entire sheets of intact cells (Figure 2: A1). To be certain this was not due to an absence of SDS in the sonication buffer or the low speed (2000 *g*) at which the samples were centrifuged during the wash steps, cells were washed with IP buffer or in SDS lysis buffer (1% SDS, 50 mM Tris, 10 mM EDTA, pH 8.0) [13], [16] and centrifuged at either 2000 *g* or 12,000 *g* for 5 min at 4°C. Cell pellets were resuspended in their respective buffers then 2.5 µl of each suspension was examined under magnification. No effect of buffer or centrifugation speed was observed (data not shown).

With the discovery that ChIP lysis buffers fail to lyse formaldehyde-fixed SMC, we asked the following questions: at what point during sonication are the cells lysed, does intensity of sonication affect cell lysis, and does the intensity or duration of sonication affect the recovery of sheared DNA in the sample supernatant after clearing by centrifugation? Fixed SMC suspended in IP buffer were spotted onto slides before sonication (Figure 2: A2), or after being sonicated for 15 or 30 min at 200, 250 and 300 W (Figure 2: B – G2). DNA was extracted via PCIA from both the supernatant and the debris pellet of an unsonicated sample (0 min) or from samples sonicated for 30 min at each intensity; DNA concentrations were determined using the NanoDrop and graphed as a percentage of the total recovered per sonication intensity level (Figure 2H). Unsonicated SMC remained intact in large sheets even after sitting in IP buffer for more than 24 h (Figure 2: A2). Surprisingly, sheets of cells were still present when samples were sonicated for only 15 min regardless of the intensity used (Figure 2: B, D and F1). Sonication for 30 min at 300 W was required to pulverize the cell sheets and most nuclei; clumps of cellular debris (Figure 2: G1, black arrowheads), a few intact nuclei and intact nucleoli (Figure 2: G2, white arrows) were recognizable. Given the microscopic observations, the sample fraction that contained the most DNA could be predicted. Centrifugation of unsonicated cells caused nearly a complete loss (97.6%) of DNA in the “debris” pellet (Figure 2H). As the intensity of sonication increased, and thus the degree of cellular damage, the percentage of DNA present in the supernatant fraction increased to 79.4% (300 W). The percentage of DNA recovered in the supernatant could not be increased by extending sonication of fixed SMC to 40 min at 300 W (data not shown).

**Figure 3:**
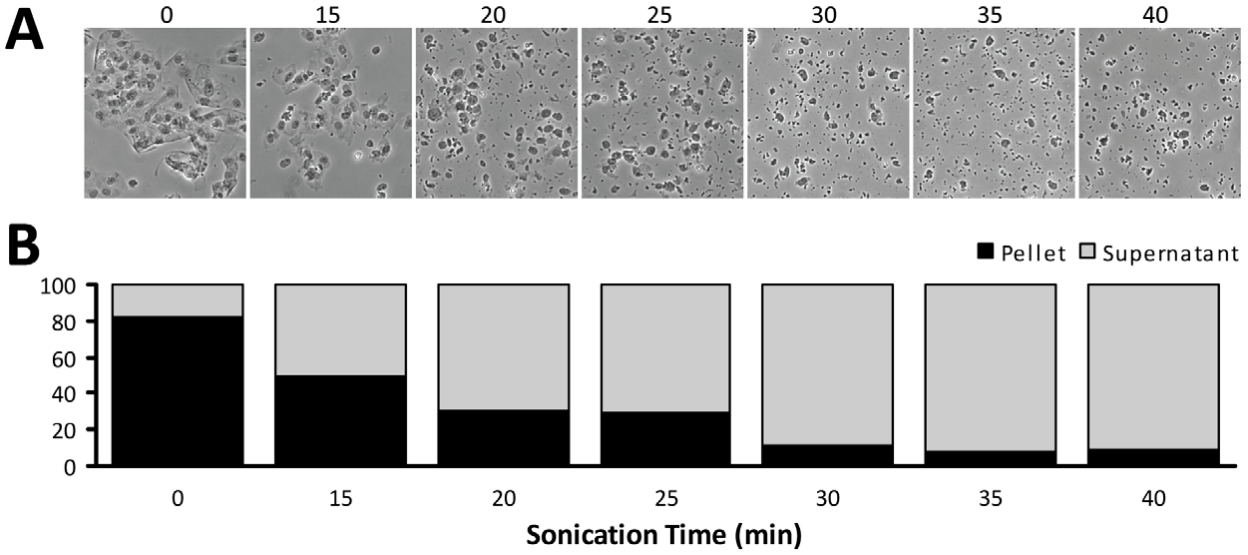
Effect of Sonication Time on Cell Integrity and DNA Recovery from Human VEC. Fixed vascular endothelial cells (VEC) suspended in IP+PPI buffer were spotted onto slides (2.5 µl) before sonication (0 min), or after being sonicated for 15 – 40 min at 300 W (**A**). Samples were viewed with phase-contrast light microscopy; images were captured using QCapture Pro51 imaging software (Magnification: 100X). DNA was extracted via PCIA; concentrations were determined using a NanoDrop then graphed as a percentage of the total recovered per sonication time point (**B**). Large sheets of intact VEC were present in the unsonicated sample (0 min). As sonication time increased, cell integrity decreased coincident with an increase in DNA recovered from the sample supernatant. After 30 min of sonication, the cell nuclei appeared to be pulverized; sonication at 35 and 40 min had no additional affect on the visual appearance of the sample and increased the recovery of DNA by only 2%.

Given that every cell type may behave differently, we tested our developing sonication protocol on a second cell type. Fixed human VEC were used to perform a sonication time course at an intensity of 300 W. Though VEC appeared to be more susceptible to cellular damage during scrapping and washing (17.5% of total DNA was present in the supernatant after centrifugation of unsonicated cells, Figure 3B), Figure 3A shows that large sheets of intact cells are still present in unsonicated VEC suspensions. As sonication time increased, cellular integrity decreased through 30 min (Figure 3A) and the ability to recover sheared DNA in the supernatant increased to 89.2% at 30 min (Figure 3B). As was the case for SMC, sonication of VEC for more than 30 min at maximum intensity failed to improve DNA recovery.

Based on the results shown in Figures 1, 2 and 3, we adopted the following parameters to shear DNA: freshly harvested cells were sonicated for 30 min at 300 W (maximum intensity for the Bioruptor XL instrument) in IP+PPI buffer. The aforementioned sonication conditions were optimized using an instrument that allowed for the simultaneous sonication of up to 24 samples. We recognize that sonicating DNA samples individually presents an avenue of introducing technical assay error that this work is unable to address. We also recognize while the two cell types tested herein behaved similarly in our hands, other cell types may behave differently in response to sonication. However, the approach we have taken to validate a mechanical DNA shearing protocol should be applicable regardless of the instrument or cell type used. One should perform a sonication time course, visually inspect the uncleared suspension after sonication to determine the point at which cell disruption has taken place, extract DNA from both the supernatant and pellet fractions after clearing to identify the point of maximum DNA recovery in the supernatant, and subject a portion of the chromatin preparation to agarose gel electrophoresis in order to confirm that the targeted DNA fragment sizes are being achieved.

### Chromatin immunoprecipitation

Because the actual chromatin immunoprecipitation portion of the assay procedure is fairly standard across protocols, regardless of whether agarose or magnetic beads are used, we did not critically evaluate this process. Our guiding protocols [7], [13]-[16] used protein A agarose beads with or without [7] salmon-sperm DNA to pull down the antibody-antigen complexes; we used the beads pre-blocked with salmon-sperm DNA *a priori* in order to maximize our signal to noise ratio. The primary difference, and a major advantage of one group's [7] protocol over the other's [7], [13]-[16], was the use of chelex-100 instead of PCIA to extract the final ChIP product in preparation for rtPCR. We found the chelex-100 DNA extraction process to be rapid and easy to perform with the added benefit of being reasonably inexpensive but the resulting sample was too dilute to evaluate sample shearing via agarose gel electrophoresis or to quantify using nanospectrophotometry (see Figure S4). In contrast, DNA extraction with PCIA was extremely time-consuming, required multiple tube changes and contained many opportunities to lose some, if not all, of the precious ChIP sample but the final volume of the sample could be smaller and more concentrated. We chose to continue using chelex-100 DNA extraction in our ChIP protocol.

### Quantification of sheared DNA purified from ChIP reactions

Because of the inherent difficulty in determining accurate cell numbers or in quantifying total DNA before experimental samples are divided into IP reactions, it is critical to accurately quantify the ChIP DNA product before rtPCR is performed. A fluorescence-based assay with PicoGreen® dsDNA dye (Invitrogen) has been used previously to quantify ChIP DNA samples [14]-[16]. The validity of this approach assumes that a linear dilution series of sheared DNA (**Ω**; harvested and prepared like ChIP samples) would maintain linearity in parallel to the reference standard (lambda DNA; **λ**) provided with the Quant-iT™ PicoGreen ® reagent kit (Invitrogen). If this was not the case, a suitable reference DNA solution would have to be generated. When this assumption was tested (see Appendix S2) it was apparent that PicoGreen-bound sheared dsDNA was detectable in a linear manner but the resulting curve was not parallel to, and thus not recognized in a manner similar to, the λ DNA reference standard (see Figure S2A). It was therefore clear that a Ω DNA reference solution would have to be used when performing the PicoGreen assay on ChIP DNA samples.

Two sources of SMC sheared DNA were used to generate the reference standard and validate the assay: 1) dedicated cells grown under similar experimental conditions then fixed, sheared, cleared and extracted using PCIA, or 2) PCIA extraction of the supernatant collected from Mock-IP ChIP reactions. PCIA extracted DNA was used (instead of Chelex-100 extraction) because it allowed for the degree of concentration required to achieve the mass range desired in the reference DNA dilution series (see Figure S2A). No detectable assay variability was apparent when a linear dilution series of sheared DNA from a single chromatin preparation (source #1) was assayed multiple times or when dilution series from multiple chromatin preparations (source #2) were assayed individually to assess the effect of shearing event (see Figures S2B and S2C, respectively). From these results, we conclude that a single preparation of Ω DNA, generated from either source, can be used as a reference standard in multiple PicoGreen assays against which ChIP DNA samples of unknown concentrations and from multiple experiments may be compared.

### Quantitative real-time PCR on quantified ChIP DNA

Normalizing ChIP samples to DNA concentration ensures that rtPCR is performed on the same mass of precipitated DNA from specific, positive and negative control IP reactions as well as from the Total DNA control sample. At the very least, including such normalization will minimize assay variation associated with differences in background fluorescence that exists when SYBR Green (an indiscriminate binder of dsDNA) is used to perform rtPCR. In addition, we predict that DNA normalization may also unmask hidden treatment effects or eliminate data artifacts.

To determine whether performing rtPCR on a specific mass per reaction or on a specific volume of the ChIP sample per reaction affected the final rtPCR data or its interpretation, chromatin was prepared from two experiments in which rat SMC were cultured with PDBF-BB (30 ng/ml) or its vehicle for 24 h then ChIP was performed with anti-SRF or anti-AcH4 antibodies. Chelex-extracted ChIP and Total DNA samples were then quantified using the PicoGreen assay. The CArG-box region of rat *ACTA2* was amplified using either 1 ng/reaction or 6.25 µl/reaction (e.g. 25% of the rtPCR reaction volume; the maximum percentage recommended [7]). Based on previously published data [16], [18], we predicted that PDGF-BB would cause a decrease in SRF binding to *ACTA2* promoter CArG boxes concomitant with a decrease in histone 4 acetylation because PDGF-BB suppresses *ACTA2* gene expression [22].

**Figure 4:**
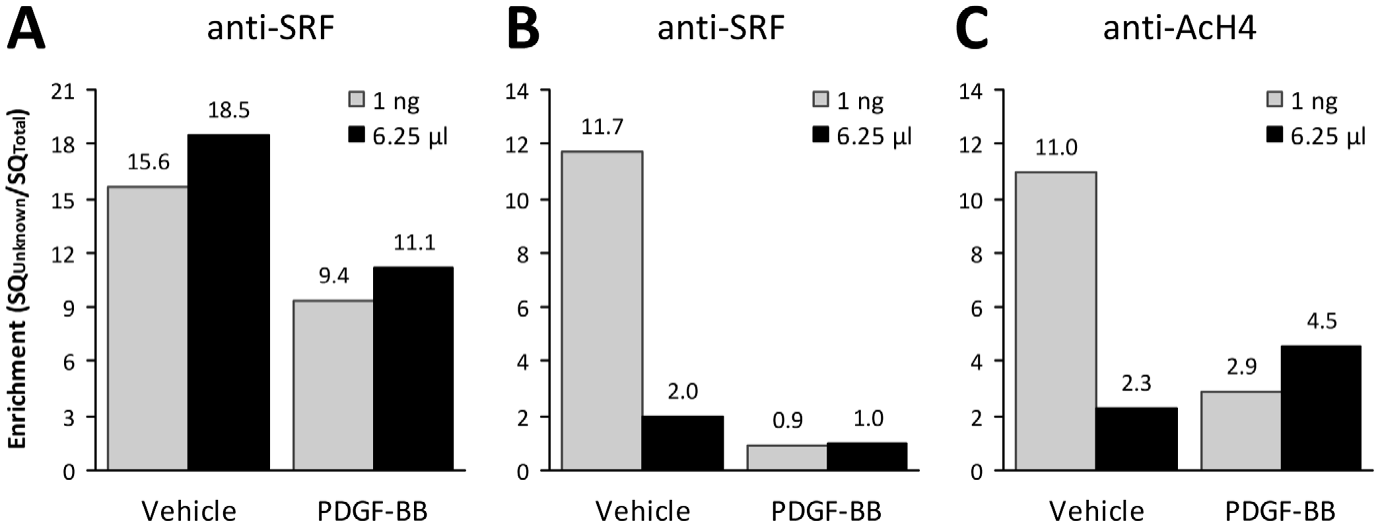
Examples of rtPCR Data Generated Using Quantified or Unquantified DNA from Specific ChIP Reactions. ChIP was performed with anti-SRF (**A**, **B**) or anti-AcH4 (**C**) antibodies on chromatin preparations harvested from two experiments in which rat SMC were cultured as described and treated for 24 h with either PDGF-BB (30 ng/ml) or its vehicle. The DNA was extracted using chelex-100 and quantified using the PicoGreen protocol. Real-time PCR was performed with either a specified mass of DNA per reaction (1 ng) or with a specified amount of ChIP sample per reaction (6.25 µl; 25% of the final reaction volume) using primers that detected the CArG-box region of the smooth muscle α-actin (*ACTA2*) promoter (bases –47 to –193 relative to the transcriptional start site). Enrichment (**A** – **C**) was determined by dividing the starting quantity (SQ) for the specific IP by the SQ for the Total DNA control. The results illustrate three different scenarios affecting the interpretation of how PDGF-BB impacts *ACTA2* gene expression: in the first experiment (**A**) there was no effect of method (a 40% decrease in SRF interaction was detected with both methods); in the second experiment, using quantified DNA magnified the detected degree of change (a 92% versus a 50% decrease) in SRF interaction with the CArG-box region of the *ACTA2* promoter following PDGF-BB treatment (**B**) and actually reversed the results (**C**) and thus the interpretation of how PDGF-BB impacts histone 4 acetylation (a 74% decrease versus a 49% increase).

In the first ChIP experiment (Figure 4A), a 40% decrease in SRF interaction with the *ACTA2* promoter was detected regardless of whether DNA normalization took place. Likewise, no effect of DNA normalization was apparent when rtPCR was performed on anti-AcH4 ChIP DNA (data not shown). However, in the second experiment (Figure 4B, 4C), quantifying the ChIP DNA unmasked a greater magnitude of change (92% versus a 50% decrease) in SRF interaction with the *ACTA2* promoter and completely reversed the interpretation of how PDGF-BB affects the level of *ACTA2*-associated Histone 4 acetylation (a 74% decrease versus a 49% increase) following treatment of SMC with PDGF-BB. DNA normalization also decreased variability in the Mock–IP data: across these two experiments enrichment values ranged from 0.16 – 0.25 with a coefficient of variation of 19.4%. In contrast, when PCR was performed on 6.25 µl of Mock–IP DNA, the range of relative enrichment was 0.01 – 0.34 with a coefficient of variation of 112%. Given the dramatic impact that DNA normalization had on the results of the second rtPCR experiment and on the variability of the Mock–IP negative control, we recommend using quantified DNA prior to performing rtPCR on ChIP samples.

### Additional assay considerations

#### Cell Harvest for the ChIP assay

The established protocol [13]-[16] that we used to harvest cells for the initial experiments presented herein was time consuming and labor intensive. It required multiple sample washes with multiple buffers before chromatin shearing took place (e. g. washing cells on the dish and in centrifugation tubes with DPBS, washing and incubating cell pellets in an NP40-based buffer to lyse the plasma membrane then switching to an SDS-based buffer to lyse the nuclei and release the chromatin for shearing). After sonication, another buffer containing TX100 was used during the ChIP step of the assay. To simplify the assay, we chose to follow a protocol that required the same number of wash steps but used a single IP “lysis” buffer containing NP40 and TX100 but not SDS [7] for the cell lysis, sonication and ChIP steps. Regardless of the protocol used, a substantial portion of the cells were left behind in the culture dish or lost during the transfer of cell pellets from large centrifuge to microcentrifuge tubes. In addition, it was apparent many times that resuspending cell pellets in detergent-based buffers resulted in bubble formation that would trap clumps of “insoluble material” [7] in the pipet tips. Because there may be times when the source is scarce, a substantial loss of sample during these washes could be the difference between assay success and failure. Our discovery that the “insoluble material” was actually sheets of intact cells and that the lysis buffers used in either assay actually failed to lyse formaldehyde-fixed rat SMC or human VEC under our conditions, resulted in a time- and sample-saving modification to our cell harvest protocol. After a single wash with DPBS to remove media containing formaldehyde and glycine from the culture dish, the cells were harvested by scraping directly in IP buffer. This change decreased the amount of time it took to prepare the samples for sonication and it decreased the amount of cells left behind on the culture dish and on the sides of centrifuge tubes. Changing to low-retention tips helped to further decrease sample loss caused by detergent bubbles trapped in pipet tips.

#### Chromatin preparation allocation for ChIP reactions

Determining how to aliquot the chromatin preparation into reactions presented a problem. Ideally, each ChIP reaction should contain the same amount of starting material. However, some protocols suggest that a target of 10 – 25 µg of DNA per reaction is ideal, whereas others suggest the DNA equivalent of a given number of cells is ideal. Some individuals even aliquot the chromatin preparation based on its estimated protein concentration. Increasing the number of culture dishes per treatment within an experiment in order to more accurately quantify cells before harvesting them or to increase the available DNA for quantification after harvesting can be cumbersome and potentially a limiting factor if insufficient sample material is available. We felt that inclusion of any step that required quantification of cells or DNA before the ChIP assay was performed would increase the complexity of the assay and the time frame in which it could be completed, thereby defeating one of our primary objectives. For these reasons, we chose to aliquot the final preparation into reactions containing the chromatin equivalent of approximately two million cells based on the estimated cell density (determined as described in Appendix S1) of the most confluent culture dish within an experiment. We felt confident that DNA shearing would be uniform across treatment dishes, regardless of density variation, and we felt that normalizing to DNA concentration *before* performing rtPCR would correct for loading differences present at the start of the ChIP assay.

#### Evaluation of the positive control ChIP target

A high degree of histone 4 acetylation is typically associated with active transcription [23]. For this reason, anti-AcH4 was initially used as the positive control in our ChIP experiments. However, acetylation is variable and susceptible to change during the process of sample harvesting [4]. Therefore, we switched to using RNA polymerase II (Pol II) as our positive control ChIP target [4], [7], [9]. To demonstrate the effectiveness of Pol II as a positive control target, rtPCR was performed on human VEC chromatin from Pol–IP and Mock–IP ChIP reactions using primers that targeted two regions of DNA upstream from the human Fibronectin 1 (*FN1*) transcriptional start site (see reference [19] supplemental material). The first set of primers amplified part of the active promoter region (bases –182 to –445; *FN1* proximal promoter) and the second set of primers amplified a region that is predicted to be transcriptionally silent (bases –1852 to –1960). Figure S5 (also see reference [19] supplemental material) shows that association of Pol II with the proximal *FN1* promoter region was 10 times greater than it was with the upstream DNA. Although a significant enrichment for Pol II was detected when the upstream DNA was targeted with rtPCR, the level of enrichment was identical to that detect by PCR performed using the Mock-IP reaction and primers targeting the proximal promoter region. This demonstrates two things: first, Pol II is an excellent positive control for the ChIP assay; and second, amplifying a region of DNA far removed from the region of interest helps to define the true level of noise within the assay system.

### Conclusion

We have presented herein, the approach taken to validate a quantitative QUICK ChIP assay that can be completed in nine bench hours over two days (the bench protocol is provided in Appendix S3). This process revealed several key considerations for a successful outcome to the ChIP assay. We discovered that IP lyses buffers fail to lyse SMC or VEC fixed with formaldehyde under our conditions. Therefore, it was critical that optimization of the DNA shearing conditions included a visual inspection of the sonicated sample to confirm that the fixed cells had been pulverized along with extraction of DNA from both the supernatant and pellet of the cleared sample after sonication to confirm maximal recovery of DNA. In our hands, achieving maximal cell disruption and DNA recovery required high intensity sonication for at least 30 minutes. Quantifying the ChIP samples using PicoGreen dsDNA dye and a sheared DNA reference standard enabled us to normalize ChIP and Total DNA concentrations prior to performing rtPCR. Doing so improved the quality of the rtPCR data.

## Supporting Information

Figure S1 **Effect of Cell Density on Rat SMC DNA Shearing Efficiency.** Suspensions estimated to contain 1.0 – 3.7×10^6^ cells in 300 µl of SDS lysis buffer were sonicated for 15 cycles of 30 sec ON and 30 sec OFF at 300 watts. Cross-linking reversal was performed before the sheared DNA was purified using the PCIA protocol; 20 µl/lane was loaded into a 1% agarose gel and subjected to electrophoresis for 90 min at 95 volts; a 100 base-pair DNA marker (M; HyperLadder II, Bioline) was used to determine the DNA fragment size range. Approximate DNA concentrations ([DNA]) were determined using a NanoDrop spectrophotometer. For the range tested herein, cell density had no apparent effect on the efficiency of DNA shearing by sonication.
(TIF)

Figure S2 **Validation of the PicoGreen® dsDNA Assay Using Sheared DNA from Formaldehyde-Fixed Rat SMC.** Reference standard stock solutions (20 ng/µl) and a dilution series (10 – 0.01 ng/µl) of the lambda (**λ**) DNA and of PCIA-extracted sheared (**Ω**) DNA purified from dedicated cell cultures (**A, B**) or from Mock-IP reaction supernatants (**C**) were prepared in TE buffer. Both λ and Ω DNA standard curves were detected in a linear manner when quantified with PicoGreen® (**A**). However, the level of PicoGreen® fluorescence detected in the PCIA-extracted Ω DNA standard curve, relative to the λ DNA reference standard, was substantially less despite the fact that the starting DNA concentrations were the same when quantified using a NanoDrop. When one Ω DNA preparation (**A**) was used as the reference standard in multiple assays over time (**B**), no assay drift was observed. Likewise, when Ω DNA reference standards were generated from multiple chromatin preparations and compared across assays (C), no assay drift was observed.
(TIF)

Figure S3 **Examples of PCR Data from Specific, Positive and Negative Control ChIP reactions.** Chelex-extracted DNA from a specific ChIP reaction (**AB–IP1**; anti-SRF) and from the positive (**Pol–IP**) and negative (**Mock–IP**) control reactions were quantified using the PicoGreen assay described herein (see Table S1). Real-time PCR was performed on 2 ng of DNA with primers that flanked the CArG-box region (–47 to –193 relative to the transcriptional start site) of the smooth muscle α-actin promoter [14], [16], [18], [21]. Enrichment was calculated as the Starting Quantity (SQ) for AB–IP1, Pol–IP or Mock–IP divided by the SQ for the Total DNA control. Enrichment of both SRF (AB–IP1) and the RNA polymerase II (Pol–IP) at the promoter was demonstrated.
(TIF)

Figure S4 **Sheared Chromatin Extracted from Rat SMC with Chelex-100 or with Phenol:Chloroform:Isoamyl Alcohol.** Supernatant recovered from experimental Mock–IP negative control reactions (A, V) were split for DNA extraction using either chelex-100 (50 µl, a volume representative of a typical ‘Total DNA’ control extraction) or PCIA (350 µl, a volume more constant with a typical PCIA extraction) according to the procedures described within the methods. DNA concentrations (**A**) were determined using the NanoDrop then either 20 µl (Chelex samples) or 2 µg (PCIA samples) of DNA were electrophoresed in a 1% agarose gel for 90 min at 95 volts (**B**). The dilute nature of the samples extracted with chelex-100 made it difficult to evaluate the degree of chromatin shearing via gel electrophoresis. In contrast, the PCIA extraction protocol allows for greater concentration of the DNA sample, thus making it possible to analyze the effectiveness of the sonication protocol via gel electrophoresis.
(TIF)

Figure S5 **Evaluation of RNA Polymerase II as the ChIP Positive Control Target.** Real-time PCR was executed using quantified DNA (2 ng) from positive (**Pol–IP**) and negative (**Mock–IP**) control IP reactions performed with human VEC chromatin. The primers either amplified part of the active Fibronectin 1 promoter region (bases –182 to –445; *FN1* proximal promoter) or amplified a region that is predicted to be transcriptionally silent (bases –1852 to –1960) approximately 2 kb upstream from the *FN1* transcriptional start site (see reference [19] supplemental material). Data were analyzed as SQ of Pol–IP or Mock–IP divided by SQ for Total DNA and are presented as relative enrichment. SigmaStat software was used to test statistical differences; pair-wise comparisons were made using a student's t-test (Pol–IP) or a Rank Sum test (Mock–IP). ** *p* = 0.002; ***p<0.001, *n* = 6.
(TIF)

Table S1 **Examples of ChIP and Total DNA Sample Concentrations Determined Using the PicoGreen® dsDNA Assay.**
(DOC)

Appendix S1 **Estimating Cell Number & Evaluating Cell Density Effect on Sonication Shearing Efficiency.**
(DOC)

Appendix S2 **Validation of the PicoGreen® dsDNA Assay with Sheared DNA from Formaldehyde-Fixed Cells.**
(DOC)

Appendix S3 **Quick ChIP Reagent List and Bench Protocol.**
(PDF)
