# Supplementary figures and images for: Chromatin Immunoprecipitation (ChIP): Revisiting the Efficacy of Sample Preparation, Sonication, Quantification of Sheared DNA, and Analysis via PCR
Source: PLoS One. 2011 Oct 25;6(10):e26015. doi: 10.1371/journal.pone.0026015 (PMC3201960; doi:10.1371/journal.pone.0026015)

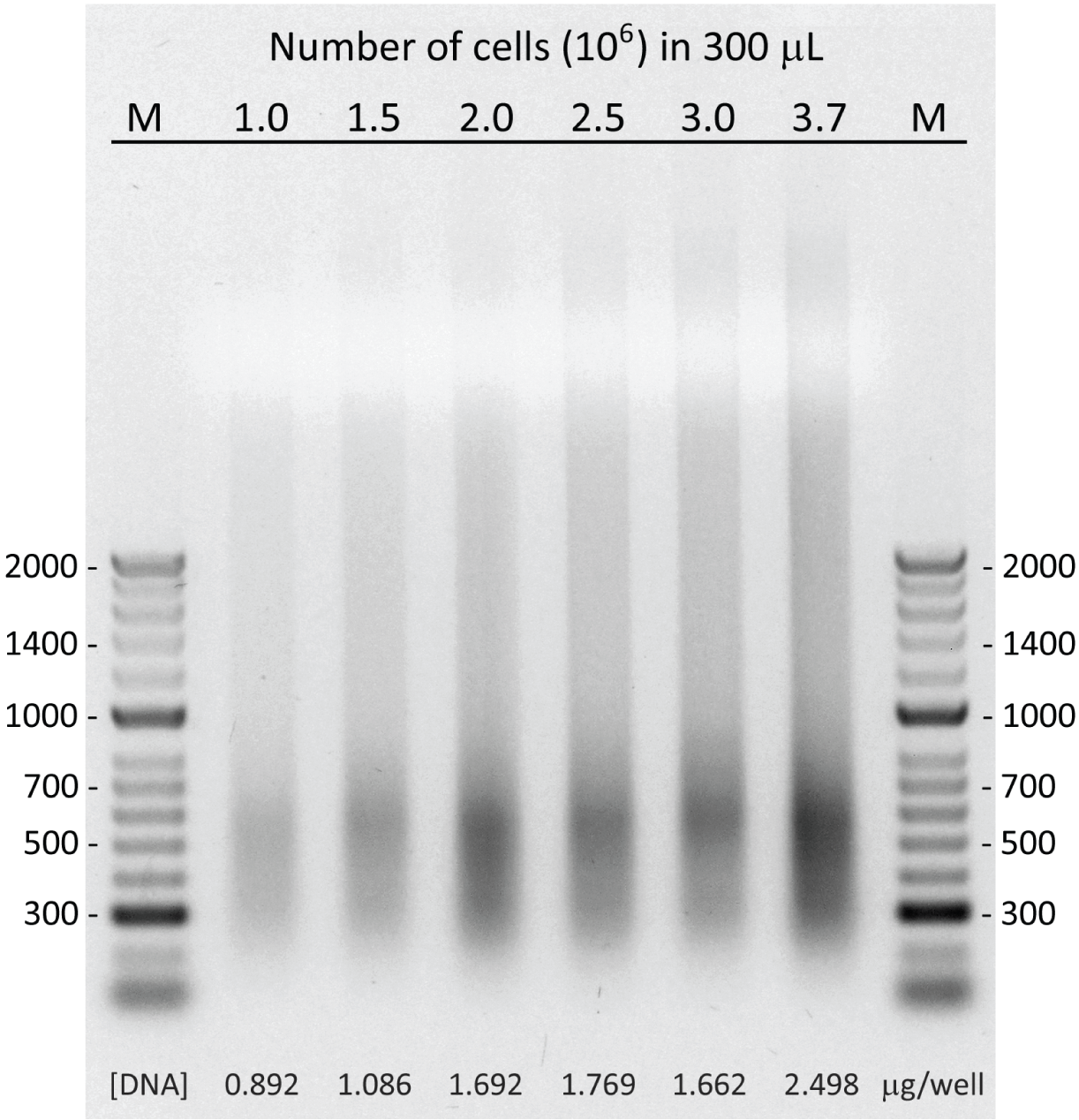

Supplement: Figure S1 — Effect of Cell Density on Rat SMC DNA Shearing Efficiency. Suspensions estimated to contain 1.0 – 3.7×10^6^ cells in 300 µl of SDS lysis buffer were sonicated for 15 cycles of 30 sec ON and 30 sec OFF at 300 watts. Cross-linking reversal was performed before the sheared DNA was purified using the PCIA protocol; 20 µl/lane was loaded into a 1% agarose gel and subjected to electrophoresis for 90 min at 95 volts; a 100 base-pair DNA marker (M; HyperLadder II, Bioline) was used to determine the DNA fragment size range. Approximate DNA concentrations ([DNA]) were determined using a NanoDrop spectrophotometer. For the range tested herein, cell density had no apparent effect on the efficiency of DNA shearing by sonication. (TIF) [file pone.0026015.s001.tif]

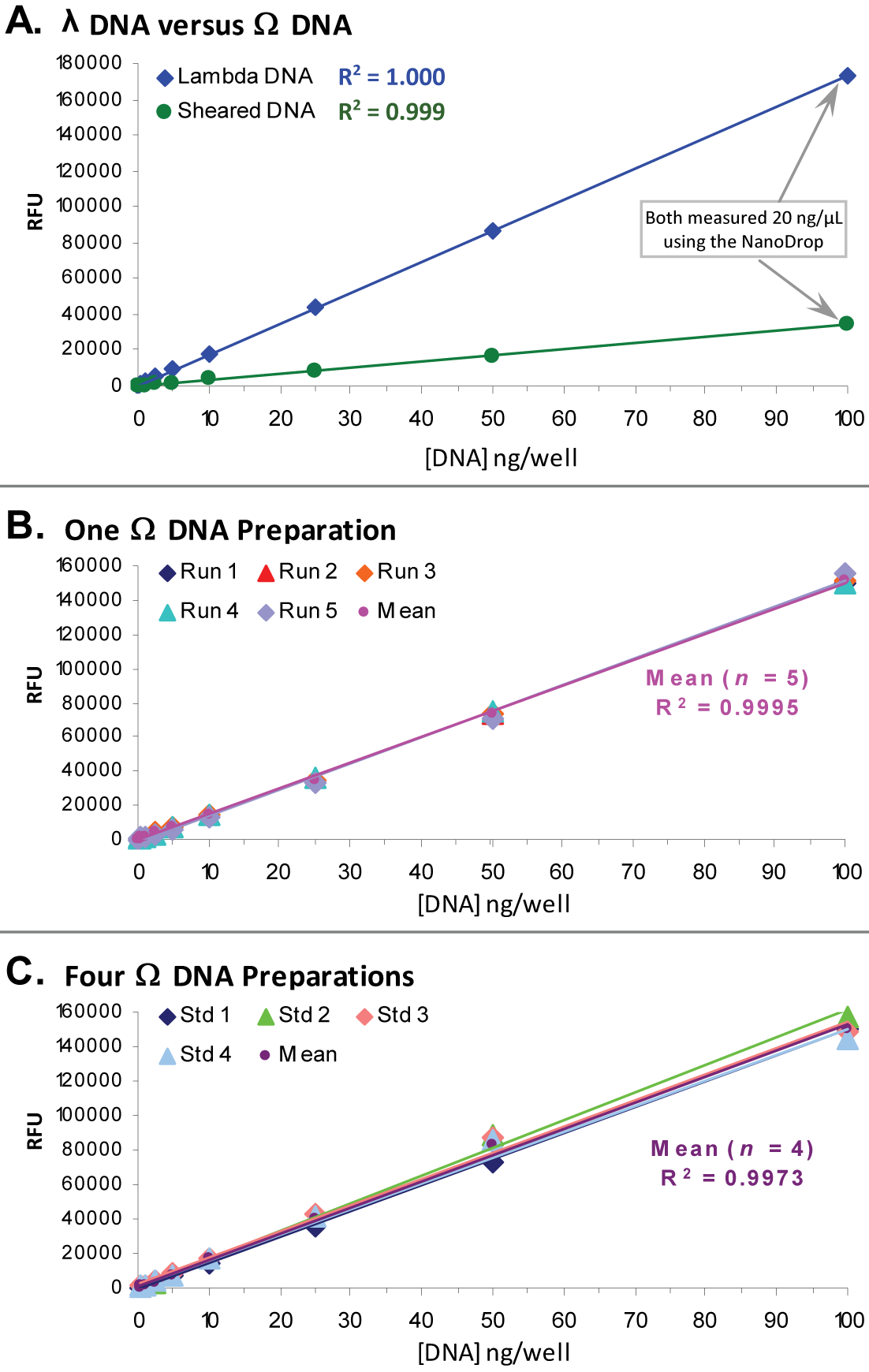

Supplement: Figure S2 — Validation of the PicoGreen® dsDNA Assay Using Sheared DNA from Formaldehyde-Fixed Rat SMC. Reference standard stock solutions (20 ng/µl) and a dilution series (10 – 0.01 ng/µl) of the lambda (λ) DNA and of PCIA-extracted sheared (Ω) DNA purified from dedicated cell cultures (A, B) or from Mock-IP reaction supernatants (C) were prepared in TE buffer. Both λ and Ω DNA standard curves were detected in a linear manner when quantified with PicoGreen® (A). However, the level of PicoGreen® fluorescence detected in the PCIA-extracted Ω DNA standard curve, relative to the λ DNA reference standard, was substantially less despite the fact that the starting DNA concentrations were the same when quantified using a NanoDrop. When one Ω DNA preparation (A) was used as the reference standard in multiple assays over time (B), no assay drift was observed. Likewise, when Ω DNA reference standards were generated from multiple chromatin preparations and compared across assays (C), no assay drift was observed. (TIF) [file pone.0026015.s002.tif]

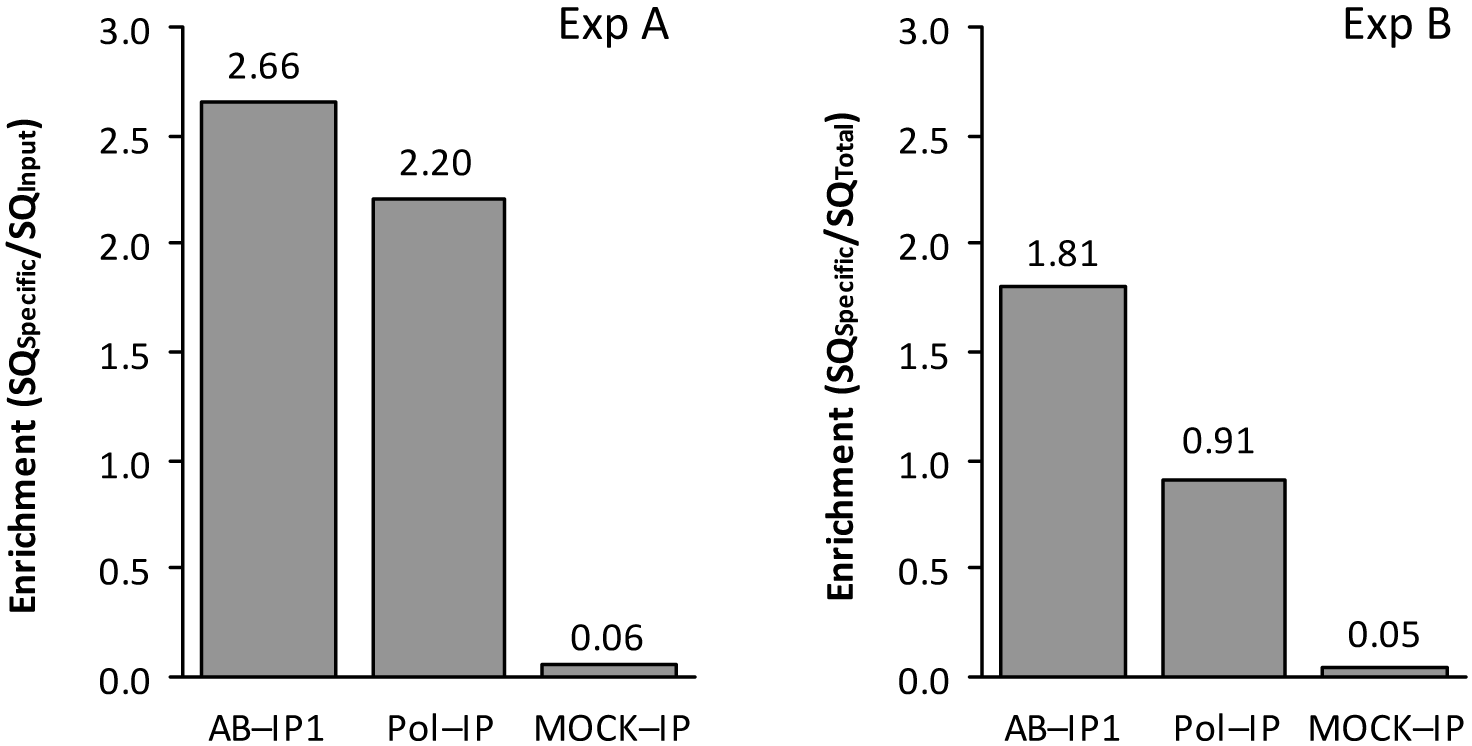

Supplement: Figure S3 — Examples of PCR Data from Specific, Positive and Negative Control ChIP reactions. Chelex-extracted DNA from a specific ChIP reaction (AB–IP1; anti-SRF) and from the positive (Pol–IP) and negative (Mock–IP) control reactions were quantified using the PicoGreen assay described herein (see Table S1). Real-time PCR was performed on 2 ng of DNA with primers that flanked the CArG-box region (–47 to –193 relative to the transcriptional start site) of the smooth muscle α-actin promoter [14], [16], [18], [21]. Enrichment was calculated as the Starting Quantity (SQ) for AB–IP1, Pol–IP or Mock–IP divided by the SQ for the Total DNA control. Enrichment of both SRF (AB–IP1) and the RNA polymerase II (Pol–IP) at the promoter was demonstrated. (TIF) [file pone.0026015.s003.tif]

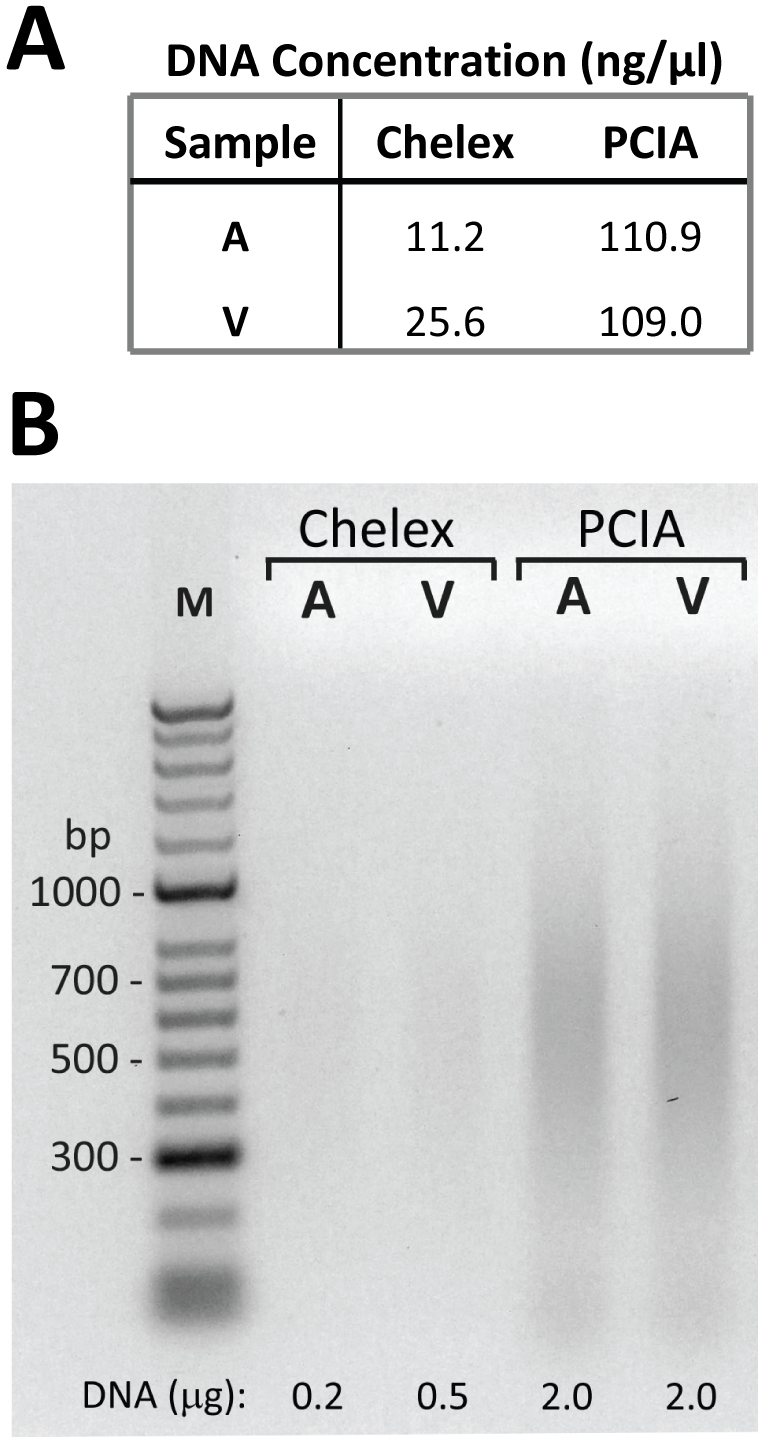

Supplement: Figure S4 — Sheared Chromatin Extracted from Rat SMC with Chelex-100 or with Phenol:Chloroform:Isoamyl Alcohol. Supernatant recovered from experimental Mock–IP negative control reactions (A, V) were split for DNA extraction using either chelex-100 (50 µl, a volume representative of a typical ‘Total DNA’ control extraction) or PCIA (350 µl, a volume more constant with a typical PCIA extraction) according to the procedures described within the methods. DNA concentrations (A) were determined using the NanoDrop then either 20 µl (Chelex samples) or 2 µg (PCIA samples) of DNA were electrophoresed in a 1% agarose gel for 90 min at 95 volts (B). The dilute nature of the samples extracted with chelex-100 made it difficult to evaluate the degree of chromatin shearing via gel electrophoresis. In contrast, the PCIA extraction protocol allows for greater concentration of the DNA sample, thus making it possible to analyze the effectiveness of the sonication protocol via gel electrophoresis. (TIF) [file pone.0026015.s004.tif]

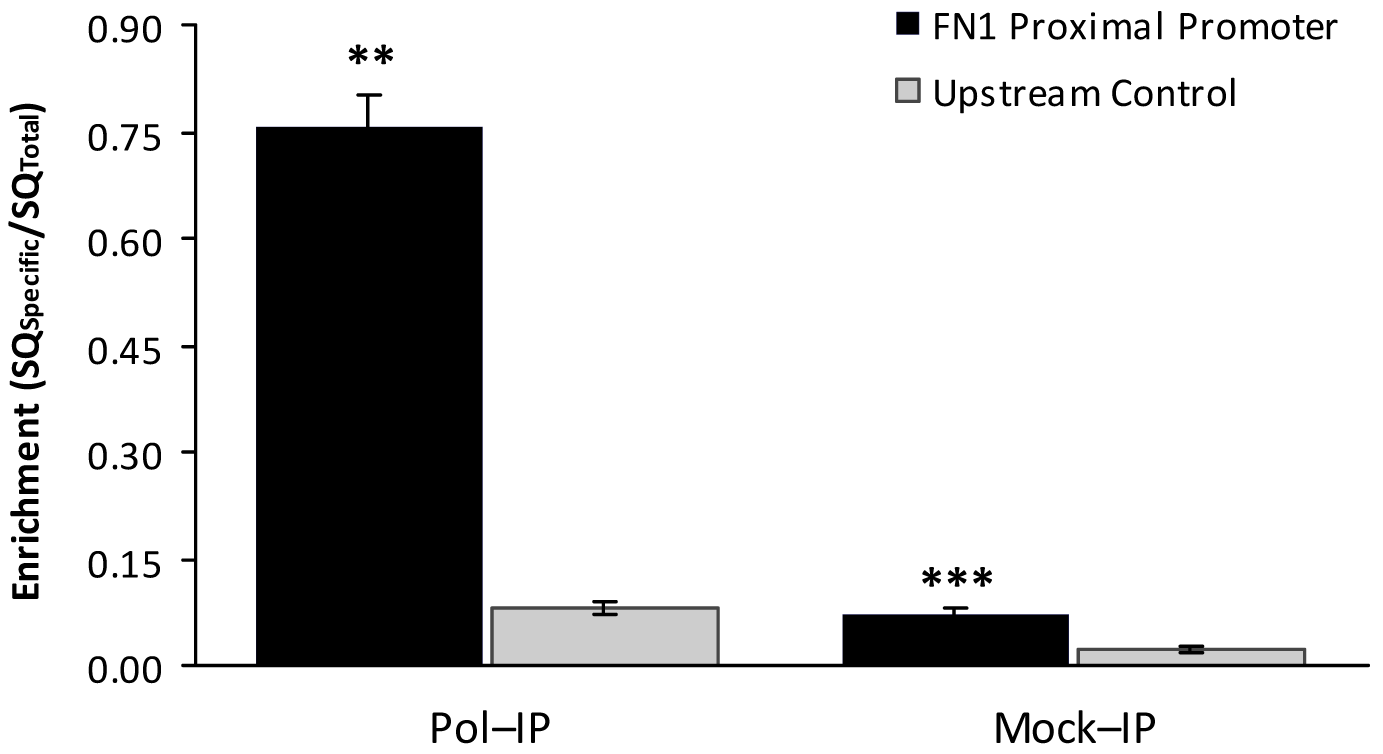

Supplement: Figure S5 — Evaluation of RNA Polymerase II as the ChIP Positive Control Target. Real-time PCR was executed using quantified DNA (2 ng) from positive (Pol–IP) and negative (Mock–IP) control IP reactions performed with human VEC chromatin. The primers either amplified part of the active Fibronectin 1 promoter region (bases –182 to –445; FN1 proximal promoter) or amplified a region that is predicted to be transcriptionally silent (bases –1852 to –1960) approximately 2 kb upstream from the FN1 transcriptional start site (see reference [19] supplemental material). Data were analyzed as SQ of Pol–IP or Mock–IP divided by SQ for Total DNA and are presented as relative enrichment. SigmaStat software was used to test statistical differences; pair-wise comparisons were made using a student's t-test (Pol–IP) or a Rank Sum test (Mock–IP). ** p = 0.002; ***p<0.001, n = 6. (TIF) [file pone.0026015.s005.tif]
