# Supplementary material for: Chromatin Immunoprecipitation (ChIP): Revisiting the Efficacy of Sample Preparation, Sonication, Quantification of Sheared DNA, and Analysis via PCR
Source: PLoS One. 2011 Oct 25;6(10):e26015. doi: 10.1371/journal.pone.0026015 (PMC3201960; doi:10.1371/journal.pone.0026015)
Supplement: Table S1 — Examples of ChIP and Total DNA Sample Concentrations Determined Using the PicoGreen® dsDNA Assay. (DOC) [file pone.0026015.s006.doc]

**Table S1. Examples of ChIP and Total DNA Sample Concentrations Determined Using the PicoGreen® dsDNA Assay.**

| **Exp^†^** | **Reaction, Target** | **[DNA] (ng/well)** | **Correction Factor** | **[DNA] (ng/µl)** | **Total DNA (ng)** |
| --- | --- | --- | --- | --- | --- |
| 1 | AB–IP1, αSRF | 4.23 | 5 | 0.85 | 170 |
| 1 | Pol–IP, αRNA Pol II | 5.17 | 5 | 1.03 | 206 |
| 1 | MOCK–IP, Rabbit IgG | 3.10 | 5 | 0.62 | 124 |
| 1 | Input*, Diluted 1:25 in diH_2_O | 1.33 | 0.2 | 6.65 | 1330 |
| 2 | AB–IP1, αSRF | 1.24 | 5 | 0.25 | 50 |
| 2 | Pol–IP, αRNA Pol II | 1.06 | 5 | 0.21 | 42 |
| 2 | MOCK–IP, Rabbit IgG | 0.74 | 5 | 0.15 | 30 |
| 2 | Total** | 42.92 | 5 | 8.58 | 1716 |

^†^Data from separate rat SMC chromatin preparations harvested on different days

*Extracted 50 µl of supernatant recovered from MOCK–IP using chelex-100

**Extracted 50 µl of starting material using chelex-100; did not dilute the sample
