# Supplementary material for: Chromatin Immunoprecipitation (ChIP): Revisiting the Efficacy of Sample Preparation, Sonication, Quantification of Sheared DNA, and Analysis via PCR
Source: PLoS One. 2011 Oct 25;6(10):e26015. doi: 10.1371/journal.pone.0026015 (PMC3201960; doi:10.1371/journal.pone.0026015)
Supplement: Appendix S1 — Estimating Cell Number & Evaluating Cell Density Effect on Sonication Shearing Efficiency. (DOC) [file pone.0026015.s007.doc]

**APPENDIX S1.**

**Estimating Cell Number & Evaluating Cell Density Effect on Sonication Shearing Efficiency.**

**Rationale**

Numerous ChIP protocols recommend DNA shearing conditions based on the density of the starting cell suspensions but the question of how to determine the starting number of cells on the plate to be harvested for the ChIP assay was not addressed. While it may be possible to count an aliquot of fixed or unfixed cells grown in suspension in advance, accurately counting fixed cells grown as attached monolayers would not be possible. Trypsinizing and counting attached cells *before fixation* was not a viable option because it would dramatically alter the targeted protein-DNA interactions. One logical solution to the problem would be to grow additional plates of cells for determining cell number or for DNA extraction and quantification. However, this would require that additional plates be harvested for every treatment in every experiment. Aside from the fact that a sufficient number of cells may not be available for this purpose, this adds, at the beginning of the assay, a significant level of complexity that we hoped to remove from the assay altogether. Therefore, we chose to estimate the number of cells/cm^2^ based on our historical cell culture data then sonicate cell suspensions at a specific cell density.

**Analyses of historical cell culture data were used to estimate number of cells per culture dish**

Rat aortic SMC have been cultured in our laboratory for several years; recording the number of cells recovered from culture flasks of specific sizes at approximate confluency levels is part of our typical cell passage routine. Analyses of this data set were performed in order to predict the number of SMC present in 150 mm culture dishes at the time of cell harvest for the ChIP assay. First, the cell density/cm^2^ (mean and standard deviation; SD) was calculated using the entire data set (*n* = 103). Outlaying data points (those greater than the mean + 2SD) were identified and eliminated; the analysis was repeated once more. A new cell density/cm^2^ (mean ± SD) was calculated for the final data set (4.16 ± 1.42 × 10^6^ cells/150 mm dish; *n* = 97) with the assumption that it represented our typical SMC culture passed at 70 – 80% confluency. Second, the data points that were excluded by the mean ± 1SD were examined (*n* = 47). Of these points 51% were greater than the mean plus 1SD and 49% were less than the mean minus 1SD. Thus, the data set was normally distributed and the values could be grouped according to an estimated percentage of confluency (<70%, 70 – 80% and >85%). Third, new means, calculated for each group (2.38 ± 0.27; 4.16 ± 0.73; and 6.49 ± 0.74 × 10^6^ cells/150 mm dish, respectively), were used to determine how much IP buffer was required to resuspend SMC pellets at a specific target density before sonication was performed.

**Effect of cell density on DNA shearing efficiency**

The cell density of a single large preparation of formaldehyde-fixed SMC was estimated as described above then aliquots of the cell suspension were diluted to 1.0, 1.5, 2.0, 2.5 and 3.0 × 10^6^ cells/300 µl (the maximum recommended volume) in SDS lysis buffer (1% SDS, 50 mM Tris, 10 mM EDTA, pH 8.1) containing 2X complete protease inhibitor cocktail (Roche Diagnostics). The diluted samples, along with an equal volume of undiluted cell suspension, were sonicated according to the chromatin shearing protocol recommended by the Bioruptor™ XL instruction manual (e. g. 15 cycles of 30 sec ON and 30 sec OFF at 300 W). After sonication, the samples were subjected to cross-linking reversal, DNA extraction using the PCIA protocol and gel electrophoresis (20 µl/lane) as described in the methods. Figure S1 shows that, for the range tested, cell density had no apparent affect on the efficiency of DNA shearing by sonication. A cell density target of 10^7^ cells/ml was chosen for future experiments.
