# Supplementary material for: Chromatin Immunoprecipitation (ChIP): Revisiting the Efficacy of Sample Preparation, Sonication, Quantification of Sheared DNA, and Analysis via PCR
Source: PLoS One. 2011 Oct 25;6(10):e26015. doi: 10.1371/journal.pone.0026015 (PMC3201960; doi:10.1371/journal.pone.0026015)
Supplement: Appendix S2 — Validation of the PicoGreen® dsDNA Assay with Sheared DNA from Formaldehyde-Fixed Cells. (DOC) [file pone.0026015.s008.doc]

**APPENDIX S2.**

**Validation of the PicoGreen® dsDNA Assay with Sheared DNA from Formaldehyde-Fixed Cells.**

Three experiments were performed to test whether sheared DNA harvested from fixed SMC could be accurately quantified using the Quant-iT™ PicoGreen® reagent kit (Invitrogen). The first experiment (**Exp 1**) was designed to determine whether a dilution series of sheared DNA would maintain linearity and be parallel to a control DNA standard (lambda DNA; **λ**) supplied with the kit. DNA was PCIA-extracted from rat SMC cultured, harvested, fixed and sheared as described in the methods. Stock solutions (20 ng/µl) of sheared (**Ω**) DNA and λ DNA were prepared in 1X TE buffer. After the concentrations were confirmed to be identical using a NanoDrop, a dilution series (10 – 0.01 ng/µl) of both solutions was prepared then assay plates were set up as described in the methods. The plate-reader protocol was set to recognize λ DNA as the assay standard with gain set at 90% on a well containing 100 ng of λ DNA (Figure S2A). The second experiment (Figure S2B) was designed to determine whether assay drift would occur when one Ω DNA dilution series (from Exp 1) was used in multiple assays over time. Experiment 3 was designed to address the question of whether using Ω DNA standard solutions purified from chromatin preparations harvested and sonicated on different days would result in significant assay drift. For this experiment, DNA was PCIA-extracted from the supernatants of three separate Mock-IP reactions and compared to the Ω DNA dilution series prepared for the first experiment (Figure S2C). For both the second and the third experiments, the plate reader protocol gain was set at 90% on a well containing 100 ng of Ω DNA.

Based on the results of the validation process, we recommend generating a serial dilution of Ω DNA ranging from 20 – 0.1563 ng/µl when performing the assay on undiluted, chelex-extracted samples that include a Total DNA sample. If the Total DNA sample is diluted, a standard range of 5 – 0.1563 ng/µl is generally sufficient to encompass the expected range of DNA concentrations. Examples of raw data generated using this assay on chromatin preparations from separate experiments are given in Table S1; the corresponding PCR results for a single promoter target are shown in Figure S4.
