# Supplementary material for: Chromatin Immunoprecipitation (ChIP): Revisiting the Efficacy of Sample Preparation, Sonication, Quantification of Sheared DNA, and Analysis via PCR
Source: PLoS One. 2011 Oct 25;6(10):e26015. doi: 10.1371/journal.pone.0026015 (PMC3201960; doi:10.1371/journal.pone.0026015)
Supplement: Appendix S3 — Quick ChIP Reagent List and Bench Protocol. (PDF) [file pone.0026015.s009.pdf]

# Appendix S3. QUICK ChIP Reagent List and Bench Protocol*

## ***Reagents Required for ChIP Assay***§

| | Reagent | Company | Cat. No. |
|---|---|---|---|
| ☐ | Formaldehyde, 37% solution; for Molecular Biology Applications | Fisher Scientific | BP531-25 |
| ☐ | Glycine Ultra, 99% pure | Fluka BioChemika (Sigma) | 50049 |
| ☐ | Dulbecco's Phosphate Buffered Saline (DPBS), sterile | Gibco–Invitrogen | 14190-144 |
| ☐ | Molecular Biology (MoBi) Grade Ultra Pure di$H_2O$ | Gibco–Invitrogen | 10977-015 |
| ☐ | Tris-HCl | Fisher Scientific | BP1532-1 |
| ☐ | EDTA (Disodium Ethylenediamine Tetraacetate) | Fisher Scientific | S312-500 |
| ☐ | Sodium Chloride (NaCl) | Fisher Scientific | S641-212 |
| ☐ | 100 % Nonidet-P40 (NP40; US Biological Corp #N3500) | Fisher Scientific | NC9983875 |
| ☐ | 100% Triton-X 100 (TX100) | USB | 22686 |
| ☐ | Chelex-100 | Bio-Rad Laboratories | 142-1253 |
| ☐ | Phenol:Chloroform:Isoamyl Alcohol (25:24:1), pH 7.8–8.2; stabilized | Acros Chemical (Fisher) | AC32711500 |
| ☐ | Proteanase K Solution (DNase free) | Roche Diagnostics | 03115887001 |
| ☐ | "Halt" Protease/Phosphatase Inhibitor Solution (100X) | Pierce (Fisher Scientific) | 78440 |
| ☐ | Protein A Agarose/Salmon Sperm DNA | Millipore | 16-157 |
| ☐ | Protein G Agarose/Salmon Sperm DNA | Millipore | 16-201 |
| ☐ | Positive Control AB: Rabbit Polyclonal anti-RNA Polymerase II IgG | Santa Cruz Biotechnology | sc-899X |
| ☐ | Negative Control AB: Nonspecific Purified Rabbit IgG | Millipore (Upstate) | PP64 |
| ☐ | 3 M Sodium Acetate, pH 7.0 – 8.0 (NaOAc) | Fluka BioChemika (Sigma) | 71196 |
| ☐ | Sodium Butyrate, 98% (NaBu) | Aldrich | 303410-5G |
| ☐ | 100% Ethanol (EtOH; pure, ***NOT*** denatured!) | Medical Storeroom | Bin 99152 |

§Does not include reagents required to pH solutions or to make and run an agarose gel

## ***Prepare the following Stock Solutions for the ChIP Assay***

| | Reagent | Tips |
|---|---|---|
| ☐ | 10% NP-40 (v/v) | • Pipet 5 ml of 100% NP40 then 45 ml of MoBi di$H_2O$ into a 50 ml tube; parafilm the cap and rock the tube on its side to dissolve the NP40 (it will take a while) |
| ☐ | 10% Triton-X 100 (v/v) | • Same as for NP40 |
| ☐ | 1 M Tris-HCl, pH 7.5 | • Add 39.4 g Tris-HCl to 200 ml MoBi di$H_2O$, pH with 10N NaOH then bring up to 250 ml |
| ☐ | 5 M NaCl | • Bring 14.62 g NaCl up to 50 ml in MoBi di$H_2O$ |
| ☐ | 500 mM EDTA, pH 8.0 | • Add 9.306 g to 30 ml MoBi di$H_2O$; pH with 10N NaOH then bring up to 50 ml |
| ☐ | 2.5 M Glycine | • Bring 9.386 g Glycine up to 50 ml in MoBi di$H_2O$ |
| ☐ | 10% Chelex 100 (w/v) | • Bring 1 g of Chelex resin up to 10 ml in MoBi di$H_2O$; store at room temperature |
| ☐ | 1 M NaBu (Optional)‡ | • Bring 5.51 g NaBu up to 50 ml in DPBS |

‡A histone deacetylase inhibitor; use fresh at 10 – 20 mM in media before fixation and in IP Buffer when histone modifications are the IP target.

### ***Immunoprecipitation (IP) Buffer [1]***

| Reagent | Dilution | [Final] | Volume (ml) | |
|---|---|---|---|---|
| 1 M Tris-HCl, pH 7.5 | 1:20 | 50 mM | 10 | 25 |
| 5 M NaCl | 1:33.33 | 150 mM | 6 | 15 |
| 500 mM EDTA | 1:100 | 5 mM | 2 | 5 |
| 10% NP-40 | 1:20 | 0.5% | 10 | 25 |
| 10% Triton-X 100 | 1:10 | 1.0% | 20 | 50 |
| MoBi di$H_2O$ | — | — | 152 | 380 |
| 100X Halt PPI† | | 1.5X | — | — |
| | | *Final Volume:* | *200 ml* | *500 ml* |

†Add fresh before use: 15 µl/ml IP Buffer

***This protocol is the result of modifications made by Pamela D. Schoppee Bortz, PhD to published methods [1-4].**

## DAY 1A: Crosslinking, Harvesting & Sonicating Cultured Cells (allow approximately 3 – 3.5 h)

➢ **Estimate the level of cell confluency before beginning the harvest. ____________ %**

1. **Fix the Protein-Chromatin Complexes**: Add **37% formaldehyde** directly to cell culture media (final concentration: 1%) then **incubate for 10 min at 37°C**.

   ☐ 280 µl/10 ml media/100 mm dish ☐ 835 µl/30 ml media/150 mm dish

   » Prechill to 4°C the 15 or 50 ml harvest tubes, 1.5 ml and 2.0 ml microcentrifuge tubes and the centrifuge/rotor/buckets

   » Aliquot and prechill IP Buffer (≈ 5 ml/150 mm dish) for **STEP 3**.

2. **Quench the Fixation Process**: Add **2.5 M Glycine** directly to the media/formaldehyde solution at a 1:20 dilution (final concentration: 125 mM) then **incubate for 5 min at room temperature (RT)**.

   ☐ 515 µl/10 ml/100 mm dish ☐ 2 × 775 µl/30 ml/150 mm dish

◆ **CRITICAL:** ***From this point forward work in the cold room (preferred) or on ice!***

3. **Wash cells**: Aspirate media then add 5 – 8 ml ice cold **DPBS** to each dish; thoroughly rinse the dish then aspirate off as much of the DBPS as possible.

   » Make the **1.5X IP+PPI Buffer** by adding **"Halt" Protease/Phosphatase 100X Inhibitor Solution** to the prechilled aliquot of IP buffer immediately before use (15 µl PPI plus 985 µl **IP Buffer**/ml)

   ☐ ______________ ml IP Buffer ☐ ________ µl PPI

4. **Harvest Cells**: Add 1.5 ml **IP+PPI Buffer** to each dish then use a cell lifter to scrape the cells off the dish.

   » Set the pipettors on 750 µl before working with the IP buffer so that buffer bubbles do not come in contact with the pipet tip filter (use low-retention tips)

   » Transfer the cell suspension to the prechilled harvest tubes then wash the dish with 1.5 ml IP+PPI and transfer the wash to the appropriate harvest tube

   » Centrifuge cells: **2,000 × *g* for 5 min at 4°C**; discard supernatant.

   » NOTE: The pellet will be loosely packed so be careful not to suck it up!

5. **Wash Cell Pellet**: Add 1.5 ml **IP+PPI Buffer** to each harvest tube; gently resuspend the cell pellet then transfer the cell suspension to a 2.0 ml microcentrifuge tube.

   » Centrifuge cells: **2,000 × *g* for 5 min at 4°C**; discard supernatant.

   » Wash the original harvest tube with 1.0 ml of IP+PPI and transfer the solution to the respective tubes containing the cell pellets.

   » Resuspend the cell pellet then centrifuge cells: **2,000 × *g* for 5 min at 4°C**; discard supernatant.

6. **Sonication**: The maximum cell density should be $1 \times 10^7$ cells/ml of **IP+PPI Buffer** ($1 \times 10^6$ cells/100 µl)

   » Optimal sonication conditions should be determined for each instrument and cell type being used.

   » Optimal DNA fragment size for transcription factors: 500–1000 bp; for Histone modifications: 300 bp

   ☐ IP+PPI/dish: ___________ µl ☐ Total Sample Volume __________ µl

**7.** Clear the lysate by centrifuging at **12,000 × *g* for 10 min at 4°C**; ***SAVE*** and combine the supernatant (containing the sheared chromatin) by treatment in a new 2.0 ml tube; mix the sample by pipetting.

**8.** Into 1.5 ml tubes, aliquot 150 – 200 µl for each specific target (**Ab–IP1…n**) plus the positive and negative control reactions; add 50 µl plus 150 µl of 100% ethanol (EtOH) into a tube labeled "**Total DNA**" then freeze at –20°C for later use in **STEP 13**.

a. Each IP reaction should contain chromatin from $2 \times 10^6$ cells (or 10 – 25 µg of DNA)

b. **Pol–IP (Positive Control):** Use an anti-RNA polymerase II

c. **Mock–IP (Negative Control)**: Use a species-matched, ChIP-grade non-specific IgG; only one negative control is required per chromatin preparation (experimental treatment) when multiple specific antibodies from the same species are used.

» Store leftover sample at –20°C or –80°C for future use. ☐ Volume per IP tube: ____________ µl

❖ **PAUSE POINT:** Can leave samples at 4°C for a few days or stored at –80°C for future use.

## DAY 1B: Antibody Incubation (allow approximately 15 – 20 min)

**9.** **Antibody Incubation**: Bring the sample volume up to 400 µl with **IP Buffer** and antibody then **incubate samples over night at 4°C with rotation** (approximately 15 – 18 h).

» For the **Pol–IP** and **Mock–IP**, use 5 µg of antibody/reaction; determine the amount of antibody used to pull down other protein targets (tubes **Ab–IP1…n**) on a case-by-case basis.

» Thaw frozen samples at 4°C

## DAY 2: Chromatin Immunoprecipitation (allow approximately 5 – 6 h)

**10.** Add 40 µl of **Protein A(G)–agarose/salmon sperm DNA bead slurry** to new 1.5 ml tubes: (use protein A to precipitate rabbit IgG and protein G to precipitate mouse IgG).

» Keep the slurry suspended while pipetting the beads, pipet them directly at the bottom of the tube then briefly centrifuge them at 2,000 × *g* to check that the bead pellets are uniform in size

» The Protein-A(G)–agarose/SSD beads are supplied as 5 ml of a 50% gel slurry suspended in 10 mM Tris-HCl, 1 mM EDTA, pH 8.0 containing 0.05% sodium azide**.** The packed beads (2.5 ml) contain 1 mg sonicated Salmon Sperm DNA, 2.5 mg BSA and approximately 7.5 mg recombinant Protein A or G.

**11.** Clear the chromatin – antibody solution by centrifuging at **12,000 × *g* for 10 min at 4°C**

» Duration of Antibody Incubation ________ h ________ min

**12.** Transfer 390 µl of the cleared chromatin to the tubes containing the Protein A(G)–agarose/SSD slurry; avoid transferring any precipitate that may contain non-specific aggregates.

» Rotate tubes at 4°C for at least 1 h ☐ Incubation Time: ____________

**13.** In preparation for **STEP 16**, precipitate the DNA from the **Total DNA** samples set aside in **STEP 8**.

» Centrifuge at **14,000 × *g* for 10 min at 4°C**; discard supernatant.

» Wash the DNA pellet with 200 µl of 70% EtOH.

» Centrifuge at **14,000 × *g* for 10 min at RT**; discard supernatant

» Dry the pellet under vacuum at RT until all EtOH is gone (≈ 20 – 30 min).

» Dissolve the dried pellet in 100 µl of 10% (w:v) Chelex-100 resin suspended in MoBi di$H_2O$

» Store the samples at RT for later use in **STEP 16**.

**14.** Centrifuge the bead slurry at **2,000 × *g* for 30 sec at 4°C**

» Transfer the supernatant from the **Mock–IP** tubes to tubes labeled "**INPUT–DNA**" (by treatment) and store at 4°C until **STEP 16**; ***SAVE*** the **Mock–IP** beads!

» Discard the supernatant from the **Ab–IP1…n** and **Pol–IP** tubes

**15.** Wash the beads: Add 1 ml of cold **IP Buffer** to all IP tubes (repeat 5 times) ☐ ☐ ☐ ☐ ☐

» Centrifuge the bead slurry at **2,000 × *g* for 30 sec at 4°C**

» *Discard the supernatant without disturbing the beads!*

» *Preheat* the Eppendorf thermomixer to 95°C; set the mixing function to 1000 RPM.

◆ **Perform the DNA isolation (STEPS 16–20) at room temperature.**

**16.** DNA Isolation using Chelex-100 Resin: Add 100 µl of 10% (w/v) Chelex-100 slurry directly onto the washed beads. Be sure to keep the slurry in suspension while pipetting.

» Vortex the samples (including the **Total DNA** tubes from **STEP 13**) briefly to mix the slurry

» Incubate in the thermomixer for 10 min at ≥ 95°C; mix at 1000 RPM.

▶ **NOTE:** The typical pH of RNase-free water is 4.5 – 5; chelex raises the pH to > 10.0

**17.** **ꔷOPTIONALꔷ** Proteinase K Treatment: Allow samples to cool to < 65°C then add 20 µg of Proteinase K

» Briefly vortex, then incubate the samples at 65°C in the thermomixer at 1000 RPM for 30 – 60 min followed by 10 min at 85°C to inactivate the enzyme.

**18.** Centrifuge the tube at **12,000 × *g* for 1 min at 4°C** then transfer the supernatant (80 µl) to a new tube.

**19.** Add 120 µl of MoBi di$H_2O$ to the Chelex/Agarose bead pellet, heat the tubes at 55°C for 5 min (to improve the DNA recovery), vortex briefly then centrifuge at **12,000 × *g* for 1 min at 4°C**.

**20.** Transfer and combine 120 µl of supernatant with the supernatant removed in **STEP 18**

» Mix and centrifuge briefly before using to ensure that no chelex is transferred to the PCR reaction.

❖ **PAUSE POINT:** Store DNA samples at –20°C; after thawing samples, mix and centrifuge briefly before using to ensure that no chelex is transferred to the PicoGreen assay or the PCR reaction

**21.** Quantify the ChIP and Total DNA samples using the PicoGreen assay then perform real-time PCR using the same concentration of DNA in each reaction.

**References**


1. Nelson JD, Denisenko O, Bomsztyk K (2006) Protocol for the fast chromatin immunoprecipitation (ChIP) method. Nat Protocols 1: 179-185.
2. Hendrix JA, Wamhoff BR, McDonald OG, Sinha S, Yoshida T, et al. (2005) 5' CArG degeneracy in smooth muscle alpha-actin is required for injury-induced gene suppression in vivo. J Clin Invest 115: 418-427.
3. Wamhoff BR, Bowles DK, McDonald OG, Sinha S, Somlyo AP, et al. (2004) L-type voltage-gated Ca2+ channels modulate expression of smooth muscle differentiation marker genes via a rho kinase/myocardin/SRF-dependent mechanism. Circ Res 95: 406-414.
4. Wamhoff BR, Hoofnagle MH, Burns A, Sinha S, McDonald OG, et al. (2004) A G/C element mediates repression of the SM22alpha promoter within phenotypically modulated smooth muscle cells in experimental atherosclerosis. Circ Res 95: 981-988.
